# Doing gender and being gendered through occupation: Transgender and non-binary experiences

**DOI:** 10.1177/03080226211034422

**Published:** 2021-09-10

**Authors:** Rebecca Swenson, Pam Alldred, Lindsey Nicholls

**Affiliations:** 1 School of Health and Social Care, 14283 London South Bank University, London, UK; 2 6122 Nottingham Trent University, Nottingham, UK; 3 School of Health and Social Care, Essex University, Colchester, UK

**Keywords:** Transgender, non-binary, genderqueer, occupational assimilation, creativity

## Abstract

**Background:**

Transgender people can face discrimination which can be reflected in and encoded by their occupational experiences. There is emerging research regarding those who are transgender but experiences of non-binary people remain under-explored.

**Purpose:**

This study considered the occupational experiences of transgender and non-binary people and how gender expression related to engagement in occupations and space.

**Method:**

Five transgender and non-binary people participated in repeat interviews, including a ‘walking interview’. Analysis was informed by new materialism.

**Findings:**

Occupational engagement can re-enforce binary understandings of gender or facilitate creative expressions of gender identity. Within normative environments, occupational participation can offer assimilation, particularly for non-binary people. Some occupations provided emancipation from binary gender norms through expression such as clothing and creative activities which provided recognition and belonging. Symbolic and personal meanings of occupations shifted when participants were able to express themselves in a way that felt authentic.

**Conclusion:**

‘Occupational assimilation’ can bring safety from scrutiny for those who are transgender and non-binary but curtails authentic expression. Occupational therapists have a role in supporting transgender and non-binary people in accessing occupations which facilitate their authentic gender expression and need to improve critical awareness of the culturally encoded binary nature of many occupations and environments.

## Introduction

‘Transgender’ is an umbrella term which describes a range of gender diverse identities (Williams, 2014) including non-binary and genderqueer which refers to those who identify with some aspects of binary identities or those who reject such binary definitions of gender and may use pronouns ‘they’ or ‘them’ (Stonewall, 2017). There is growing evidence that individuals who define as transgender or non-binary face discrimination globally across health care settings (Lo and Horton, 2016). For example, within the UK, it has been identified that transgender and non-binary people experience discrimination, health inequalities and reduced access to health services (NHS England, 2015). Whilst there has been an increase in research into these communities (Vincent, 2018a), there remains a need for continuing research into the occupational experiences and needs of these communities (Dowers et al., 2019).

To appreciate how opportunities for occupational engagement relate to identity, particularly those which are marginalised, it requires a broader understanding of the influence of factors, such as culture, society and politics (Hocking and Wright St Clair, 2011). That is, the relationship between occupation and identity is ‘situated’ in a socio-political understanding of time and place. The meaning a place has for an individual has a bearing on developing and conveying of identity (Huot and Laliberte Rudman, 2010). An occupational understanding of the lived experience of time and place has common theoretical ground with queer theory, which is also concerned with the impact of social constructions on the lives of individuals (Hammell and Iwama, 2012). Furthermore, theorists from the field of occupational science have illuminated the relationship between queer theory’s gender performativity and occupational engagement (e.g. Beagan et al., 2012). Queer theory posited that gender is a non-verbal performance constructed through the ‘*stylized repetition of acts’* (Butler, 1990: 191).

It is through this lens of socio-cultural productions of gender that the experience of individuals is understood and some view a pre-existing gender as expressed through occupational engagement (Dowers et al., 2019). This humanist understanding of the individual has shaped occupational therapy; the Royal College of Occupational Therapists (2015: 1) stating that people ‘have the capacity to transform themselves through premeditated and autonomous action’. Yet this capability for transformation to express gender identity freely for those who are transgender or non-binary is curtailed by societal attitudes. For example, people from these communities reporting that they adjust the way in which they dress and which ‘gendered’ occupations they are seen as doing for fear of harassment (Stonewall, 2018). This study explored how different occupations and the spaces that were inhabited related to the expression of gender for those who define as transgender, non-binary or genderqueer.

## Literature review

In Europe and North America, there is increasing research attention to the experiences of transgender and non-binary people, but compared to other health care fields, research into transgender occupational experiences is relatively limited.

### Occupational experiences of transgender people

Reed et al. (2010), in their study into the lived experience of an occupation, discussed how a transgender woman, through the occupation of going for walks in her local community was able to reveal her female self to others. In a subsequent study, five transgender women were interviewed about their occupations, and it was found that participants experienced loss of occupations such as swimming or undertaking a parenting role (Beagan et al., 2012). Taking a quantitative approach Avrech Bar et al. (2016) examined, through interview and three self-report measures, the relationship between gender identities and the occupations of 22 transgender women and 22 cis gendered women (i.e. those whose birth sex matched their gender identity). The transgender women were all found to have reported lower scores of occupational performance, particularly in relation to their lives pre-transition (Avrech Bar et al*.*, 2016). A significant finding of the study was that participants, following their transition, feel obliged to conform to societal expectations of their gender by participating in activities understood to be feminine in order to get recognition (Avrech Bar et al., 2016). These conclusions resonated with the findings of Schneider et al.’s (2019) study into the occupational transitions of four transgender adults, some of whom described how, post-transition, they engaged in gender binary stereotyped occupations to affirm their gender identity. The study found how gender transition correlated with changes in occupational engagement (Schneider et al*.,* 2019). Daly and Hynes (2020) explored the changing roles at work following transition and highlighted the complexity of transitioning in the workplace and the change in roles this can entail.

In a scoping review of 41 articles from differing professional and theoretical fields, Dowers et al. (2019), explored transgender people’s occupational experiences. They concluded that there were unequal occupational experiences for those who define as transgender and that adaptive strategies were employed to enable occupational participation. Significantly, the review also highlighted how occupational engagement enables transgender people to express their authentic gender identity. McCarthy et al. (2020) attended to the experiences of non-binary people within heteronormative environments and highlighted the complexity of expressing non-binary gender in normative environments.

### The role for occupational therapy

A potential role for occupational therapists in addressing occupational injustices for transgender people has been identified by Avrech Bar et al. (2016) and was the focus of Jessop’s (1993) case study of working with a ‘transsexual’ client. Whilst an understanding of transgender identities has advanced since this latter article was written, it indicated how occupational therapists are well placed to support an individual’s expression of their authentic gender (Jessop, 1993). Pope et al. (2008) highlighted the ethical issues of informed choice and professional responsibility by presenting hypothetical case studies whose choice of clothing the occupational therapists felt conflicted with social expectations. The authors presented a scenario of a transgender client who is deemed by the occupational therapist to be wearing inappropriate female clothing. Whilst the article is not critical of the hypothetical client, it highlights how occupational therapists could be seen to be arbiters of the gender binary, rather than facilitators of gender expression. This resonates with concerns that have been raised by transgender individuals when engaging with health care professionals that they feel health care professionals are unduly critical of, and attempt to ‘police’, their dress (Beagan et al., 2012).

These studies highlight how occupations could enable the authentic expression of gender identity but there remains unequal occupational opportunities experienced by transgender people. With the exception of McCarthy et al. (2020), these articles provided a binary understanding of gender and some positioned transitioning as a one off event rather than a complex, fluid process. In understanding gender transformation as binary and finite undermines the gender diversity of those who are transgender (Vincent, 2018b). This doctoral study aimed to address this lack of multiplicity by exploring how gender identity is expressed in relation to occupations and situated spaces by both transgender and non-binary people.

## Method and methodology

### Epistemological position

The research adopted a queer methodological approach which acknowledges the fluid and fragmented nature of identities (Crosby et al., 2012). Central to the theoretical position of the research is new materialism which understands that there are materials outside of language that have a bearing upon us (Alldred and Fox, 2015). These materials such as buildings, human bodies, legislation and everyday objects are considered together in what can be called ‘assemblages’, after what Deleuze and Guattari (1987) called an *agencement* or collection of things gathered together. This relational understanding of human bodies and the material realities of daily lives resonates with occupational science’s position that environmental realities such as cultural objects or social policies are implicated in our experiences of ourselves, including our gendered sense of self and, indeed, may be perceived as part of one’s self (Hocking, 2000). The qualitative methods used, which reflected this understanding, involved an evolving set of semi-structured and walking interviews as these were understood to be able to elicit individual experiences and meanings (Silverman, 2004). Like occupational therapy, new materialism emphasises embodied experience, hence we will refer not to identities as if pre-existing the material world, but as produced within and by it.

### Ethical procedure and reflexive statement

University ethical approval was granted in February 2017. An advisory group of four individuals who define as transgender or non-binary were recruited to help ensure that the research did not exploit participants and had relevance to transgender communities. As an outsider to the research population, namely identifying as cis gender, author 1 felt it was important to exercise what Vincent (2018a) referred to as an ethics of care towards transgender participants. The group advised at all stages in the research process. A journal was kept by author 1 in which observations and feelings prior to, during and after the interview were recorded. These reflexive accounts were incorporated into the analysis of the in-depth interviews with the participants.

### Research participants

Participation was open to anyone over the age of 18 years, English-speaking and based in the UK, who defined as transgender, non-binary or genderqueer, reflecting current terms for the expansive and fluid identities within the transgender communities. Snowball and respondent-driven sampling was undertaken, approaches which are understood to be appropriate when participants can be difficult to contact (Whittaker and Williamson, 2011). Adverts were circulated within lesbian, gay, bisexual and transgender (LGBTQ+) groups, venues such as libraries and online platforms.

Five participants were recruited, and this was considered to be a suitable size of study group for this type of research where the exploration of lived experiences is valued for depth and biographical contextualisation, rather than for generalisability of the findings (Emerson and Frosh, 2004). Written informed consent was obtained from all participants. One participant (Dee) defined as a transgender woman, one (Fred) as trans masculine non-binary and the remaining participants (Edie, Sam and Max) defined as non-binary and genderqueer (pseudonyms are used here). Eli contributed one interview and the others, three each.

### Interview process

With the exception of Eli, participants were interviewed three times over a 1 year period. This approach was selected to enable the capturing of a depth of data necessary to address the research question and in order to work alongside the participant in the production of data (Finlay and Evans, 2009). The length of the interviews ranged from 45 to 90 min. The initial interview was semi-structured and took place in meeting rooms near the participants’ homes. The opening question was kept suitably broad to elicit narratives regarding identity, which was ‘Can you tell me about yourself? ’and participants were asked to bring a photograph of a meaningful activity or place as a means not only of stimulating discussion but also as a way of eliciting their perspectives on their relationship to objects and places. The second, unstructured interview asked follow-up questions in order to clarify and elicit further narrative accounts, these took place in venues chosen by participants, such as cafes. The final interview was a *walking interview* whereby participants were invited to choose a route to walk with author 1 while the interview was conducted.

Walking interviews are considered to generate rich data as participants’ accounts are prompted by their associations with the environment (Evans and Jones, 2011). All but one of the four participants engaged in this (one was unable to due to an injury and instead, at the participant’s choosing, the interview was held instead in a café) and participants chose a route that was meaningful to them, such as city streets and parks.

### Analysis of data

Following discussions with the advisory group regarding data analysis, interviews were audio recorded, transcribed and data were coded by hand and in NVivo. A new materialist ontology informed the data analysis as outlined by Alldred and Fox (2015).

The first stage of analysis was reading transcripts, alongside field notes, to identify components that could be seen to constitute a gender expression assemblage (such as economic issues, workplaces and the home) within participants’ accounts. The second stage was a re-reading of transcripts shifting focus from what the components *were* to, what they *do*, namely, how they worked together to create affects and capacities such as actions and emotions (Alldred and Fox, 2015) that influenced gender expression – produced gender – for each participant. The final stage of the analysis was to understand the micropolitics of the assemblage which are understood to exist as territories which either act to consolidate (‘territorialise’), a state (such as gender) or de-stabilise, ‘de-territorialise’ and allow new ways of being and becoming (Deleuze and Guattari, 1987). For example, within an assemblage, legislation may interact with a specific environment (such as public changing rooms) to consolidate and fix binary understandings of gender.

Transcripts were sent to participants and interpretations discussed in subsequent interviews, which contributed to the inclusivity of the research as well as the validity (Finlay and Evans, 2009).

## Findings

Findings showed intra-actions within assemblages, amongst elements such as participants, the home, the workplace, legislation, hobbies and clothing which together facilitated gender expression. The analysis is presented in terms of how engagement in occupations territorialise – stabilise – binary forms of gender expression yet also create opportunities to de-territorialise, that is, facilitate new forms of gender expression.

### Territorialisation: occupations stabilising binary expressions of gender

Participants described how engagement in activities reinforced binary gender expressions. For Dee, as a transgender woman, engaging in occupations which reinforced binary gender roles was integral to her gender expression both pre- and post-transition. Before transitioning gender, when living as a man, she had felt compelled to engage in hyper-masculine activities such as white-water rafting to produce masculinity. Following her transition and being able to live openly as a woman, Dee engaged in activities which expressed her female gender identity, such as decorating her home in ‘*feminine*’ furnishings and joining a social group for women where they ‘*do all sort of social activities, go on holidays together’*. Walking was a highly valued occupation for Dee and the occupational experience of this activity shifted pre- and post-transition. It was an occupation that remained the ‘same’, but its symbolic meaning for her had changed. Pre-transition it provided Dee with respite from the masculine environment:

‘Walking was my escape. I could walk for miles and hardly acknowledge anything else, I would just be in my head and be in my world’ (Dee).

Following her transition, walking remained an important activity, but was no longer about escapism, but rather its pleasure was from ‘*enjoying the environment, just enjoying the exercise’.*

Organised group creative activities rather than freeing gender expression enforced binary understandings of gender. Max who attended dance classes explained that the lessons were structured upon male and female roles and there was ‘*no space for me’.* Likewise Fred stated that the LGBTQ+ choir he attended was not ‘*the right kind of space for me’* because ‘*voices get gendered’.* Participants also described how their gender expression was kept in check by societal attitudes. Sam and Eli discussed how occupations enabled them to express their identities and/or challenge the binary notion of gender. This was done through engaging in occupations such as wearing make-up and clothing that was either androgynous or associated with a particular gender identity. However, such occupational engagement did not come without risk; Sam discussed their experiences of wearing dresses and make-up:

[it] drew a lot of comments and even when I thought I was doing ok, I mean I know my voice isn’t particularly light or anything and I know I’m tall, I know I have broad shoulders and […] I did this for me [laughs] not anyone else. Um, but yeah [sighs] it was just a lot, especially when I really sort of put myself out there I got comments. I really didn’t like it and trying to explain it. [Sam]

Sam and Eli found such interactions *‘exhausting’* [Sam] and as a result limited their wearing of such clothing in public. Such consideration of how, when and where occupations could be undertaken was described by Fred, who defines as trans masculine non-binary, in going to the gym:

So, I go to the local gym and that has a completely gendered changing areas there’s no options given at all and the way that I currently cope with that is that I very rarely go by myself, so I go with somebody else. We both use the same changing room, strength in numbers [Fred]

Alongside changing rooms, toilets were a significant environment which enforced binary gender expressions and Fred described his ‘*terror*’ at entering a toilet and fear of having his gender queried and scrutinised. As well the impact of accessing a toilet, or not being able to, there were also wider implications of a radical adjustment to daily lives. Dee spoke of how, until she had undergone surgery, ‘*I wouldn’t go anywhere near an environment where I had to be, expose myself’* and as a consequence, did not feel she could go swimming.

The childhood home and workplace were contested sites of gender expression. Sam described how as a child they were not allowed to have toys or engage in play which was deemed feminine or even decorate their room in feminine colours. All participants, apart from Eli, discussed how the workplace inhibited their gender expression and this was particularly true of those who defined as non-binary such as Max who stated: ‘*I don’t feel I can present authentically in the work space’*. They explained how this not only created a dissonance with their gender expression, but also affected their work role:

All this stuff gets in the way of you being creative and contributing positively in your job so that’s the bit that I find inhibits me […] I do feel that um, I’m held back in how well I do the job because um, you know I’m constantly minding how am I [pause] presenting, how am I being read? Do I sit, how do I hold myself? How do I, it’s that, that constant wanting to not attract attention. [Max]

Similarly, Sam felt that they would not have got their job if they had revealed their gender identity to their employer and, in not doing so, felt that they had ‘*discarded part of [themselves]*’. Binary presentations of gender were kept in check in Sam’s workplace through the wearing of gendered uniform which further heightened their dissonance with the environment. Fred, Dee and Sam spoke of how colleagues on occasion inadvertently misgender them by using incorrect pronouns causing feelings of ‘*anger’* and ‘*grief’* [Fred].

### De-territorialisation: ‘authentic’ gender expression and occupational engagement

It was clear from the findings that engagement in occupations important to participants enabled a creative expression of self and, furthermore, recognition and belonging. Clothing choices and in some cases make-up enabled an expression of gender identity that challenged binary conceptualisations of gender:

some days I’ll dress like masculine, well however masculine is presented. But then some days I’ll just want to wear a dress and be done with it [laughs]. So, I want that to be ok, like I just want to wear a dress or a skirt or something and still be seen as a boy. [Eli]

Sam described his engagement in role playing games (RPG) – where players assume the identity of a fictional character in imagined settings – enabled a creative expression of self which is not modelled on binary tropes. As Sam commented in relation to RPG and their gender expression, ‘*you start thinking outside the box a lot more’* and this helped them to ‘*start expressing’* their gender identity within a supportive environment. Seemingly, engaging in an activity which demands imagination became a conduit for authentic gender expression.

The environmental context and the meaning it held to the individual positively influenced occupational engagement and their expression of gender. All participants described how the ‘home’ sometimes provided the only safe environment for their gender expression. As Sam explained ‘*at my home I can be myself and there’s no persona to it’.* However, this was contingent on the home being lived in either by themselves or with someone they trusted. For all participants the internet was transformative in terms of empowering gender expression. It enabled discovery and provided connections:

And I logged in and I found like-minded people who would talk to me and explain things and I got on to the chat rooms and I just lapped up all the information and gradually absorbed it all so that I realised that I wasn’t a, I was transgender, I wasn’t a transvestite. [Dee]

Of significance was how occupations enabled participants to connect with like-minded individuals, and this created, for Sam in the context of RPG a *‘home’* and a ‘*space to expand into’.* Eli described how going out for a coffee with friends who also defined as transgender provided a sense of connection, ‘*we meet up* […] *and just laugh together’.* These did not necessarily occur in identified LGBTQ+ venues and in fact largely did not, but rather spaces in which commonality and belonging were forged through activities. Dee described a transformative occasion when she first wore in public female clothes at a music event at a deserted warehouse. This was not a space specifically for LGBTQ+ people, but the event and the privacy of the venue facilitated a gathering where it was safe to express her gender identity freely and to subvert gender expectations.

## Discussion and implications

This study explored how engagement in occupations and spaces had affected the gender expression for those who define as transgender and non-binary. Its findings, supporting current research in the field (Avrech Bar et al*.,* 2016; Beagan et al., 2012; Dowers et al., 2019; McCarthy et al., 2020; Schneider et al., 2019), demonstrated how occupational engagement is critical for the expression of authentic gender identity for those who define as transgender and non-binary. Namely, engagement in occupations enabled an expression and recognition of identity and a means of connecting with others, but this was experienced differently for those who had a non-binary identity and those with a binary transgender identity. Applying a new materialist lens to the findings foregrounded how gender expression resulted from the interaction between object, subject, environment and action. Furthermore, it highlighted how there is not one volitional agent amongst this assemblage, but a collective agency (Deleuze and Guattari, 1987). For example, walking, a significant occupation for one participant, produced different affects pre- and post-transition; pre-transition, its meaning was that it served to exhaust her physically and mentally, numbing the realities of being forced to present as a man. Following her transition, when living openly as a woman, walking became restorative, in that it provided wellbeing through its connection to the environment. Developing Schneider et al.’s (2019) finding regarding occupational transitions aligning with gender transitions, this suggests that the meaning of occupation can transition alongside gender expression.

Findings reflected how transgender and non-binary people are vulnerable to microaggressions, such as staring when accessing the everyday spaces of public bathrooms and changing rooms (Stonewall, 2018). Not only does this serve to prevent the engagement in fundamental of self-care activities, namely toileting, but serves to prohibit engagement in occupations within that wider environment. Public toilets and changing rooms served to regulate movement and territorialise binary understandings of gender and in doing so regulated gender expression. Participants described how as adults there was more freedom to explore their gender identity, initiating potential in occupational transition in relation to their gender expression (Schenider et al*.,* 2019). Yet, institutional influences were evident in adulthood specifically within the workplace which did not just reflect social and cultural expectations of gender expression but affected its enforcement.

Participants described conforming to gendered expectations through engagement in activities within the work environment to avoid scrutiny and reprobation of their authentic gender identity. These included engaging in hyper-masculine social activities, wearing a uniform which contradicted their gender identity and the manner in which they engaged in activities. This was heightened for non-binary participants, for whom there is arguably less comprehension regarding such identities, who experienced questioning about the fluidity of their gender expression. They described how they censured their gender expression not only to keep safe but also to prevent questioning by others about their identity. Occupations in effect provided a camouflage from a potentially hostile environment which facilitated a compromised recognition rather than scrutiny. Such experiences could be argued to be akin to occupational alienation, whereby engagement in activities which are devoid of meaning are considered to create a ‘sense of isolation, powerlessness, frustration, loss of control and estrangement’ (Wilcock, 1998: 343). Whilst this definition holds some similarity to participants’ accounts, it does not reflect the entirety of their experiences. That is, there was a motivation, born from necessity, to undertake such activities because they provided security, through conformity. As such, the term ‘occupational assimilation’ is seemingly more apt as occupational engagement provided a recognition, although not of their authentic self, which situated participants within their environment. This appears to be similar to the experience of cultural assimilation expected of immigrants. Assimilation in the context of immigration is ’rewarded’ with acceptance by the host culture because of ‘fitting in’ (Henry et al., 2005). Passing in a binary gender culture might be likened to this and therefore the problematic occlusion of other aspects of an individual, the hiding of difference or lack of fit might be consequent.

Lines of flight are described by Deleuze and Guattari (1987) as moments of change within an assemblage and such happenings were apparent in participants’ accounts when they disrupted gender norms and were able to express their authentic identity. Engagement in important activities, and more so ones that enabled creativity, provided such instances of emancipation from binary conceptualisations of gender. For example, the alternative and fantastic world of RPG subverted gender roles for Sam and the feminine ascribed activities of Dee’s women group allowed participants to bond with one another. This was at odds to those activities such as dance classes, which are deemed to be creative but replicated binary expectations of gender. Radical gender expression can be met with disapproval and even violence, and arguably activities such as RPG enabled this to be undertaken safely. Activities also established a kinship and belonging between those who undertook them – Eli, Dee and Sam spoke of how co-occupations in shared environments in familial terms. For non-binary participants, expression was embedded within de-territorialisation; however, for Dee whilst a distinct moment of de-territorialisation, namely attending a music event in an abandoned warehouse, opened up new ways of being to identify as a woman, it was the process of territorialisation – presenting as unequivocally female and undertaking gendered activities – which stabilised and secured this identity and expression.

The research illustrated the barriers to occupational engagement particularly for those who are non-binary and navigating gendered environments. Occupational therapists are arguably well placed to address such impediments to occupational engagement, or to tackle oppression within specific environments. For example, participants highlighted how workplace environments can be hostile to, or lack understanding of, expression of gender for those who define as transgender or non-binary. Occupational therapists could be well placed to work with employers to raise awareness in terms of attitudes (such as the correct use of pronouns) and the environment (for example, the importance of gender neutral toilets).

Furthermore, there is a need to develop reflections and training on gender expression and occupation within health and social care settings and on pre-registration curricula. Further research into the occupational experiences of these communities, particularly non-binary communities would be beneficial. Research into occupational therapists’ own experiences of working with transgender and non-binary people would help develop an understanding of current and potential roles for the profession in supporting these communities.

## Limitations

The purpose of qualitative research is to develop deeper insight into complex phenomena and here, by studying the meaning of individuals’ experiences (Wu et al., 2016), we have sought to understand how occupation operates to produce and experience gender. As such, an identified limitation of this study lies not with there being small numbers of participants, but in that, given the fluidity and complexity of gender and the number of differing and intersecting identities that fall under the transgender umbrella, a larger group of participants would have enabled a broader range of transgender and non-binary people to be included. A particular tension of the approaches adopted here, is that occupational therapy embodies humanist and person-centred understandings, whereas we seek to apply a post-human approach to analysis of the ‘more than human’. As a result, we have adopted the language of identity and authenticity when this is about the experience of the participants and the language they used, but use a language of agency for material elements of the assemblages that had some force in producing their experiences of gender.

## Conclusion

This study foregrounded the occupational experiences of non-binary people and illustrated how occupational experiences can differ for those who are non-binary and those who have a binary transgender identity. Furthermore, occupations can connect transgender and non-binary people to a community but also offer ‘lines of flights’ from previous identities, roles or communities which allow for new ways of being (Alldred and Fox, 2015). However, not only did non-binary people face discrimination in the form of abuse and negation of their gender identities, the prescribed gender roles associated with occupations also served to territorialise, that is, shore up a binary gender expression. In order to avoid scrutiny, non-binary and transgender people sometimes feel the necessity to undertake occupations which conceal their identity and convey a binary gender. Such occupational assimilation may provide safety, but at the cost of creative expression and as such occupations in these instances are purposeful, rather than meaningful.

The deprivation of occupational choices for transgender, non-binary people causes an exhaustive undertaking of adaptive strategies to undertake meaningful and necessary occupations, sometimes at great emotional cost and certainly another task to do. This study adds to the growing research base and identifies key areas in which occupational therapy, a profession which aims to address social concerns through its practice with marginalised groups, could support transgender and non-binary people.

## Key findings

- Occupational assimilation can enable fitting in to hostile environments.
- Non-binary people have distinct occupational experiences to binary identities.
- Occupations can enable creative expressions of gender identity.

## What the study has added

This study contributes to research into the occupational experiences of transgender and, in particular, non-binary communities, highlighting how occupation can produce assimilation in heteronormative environments or enable creative gender expression.
